# Treatment Considerations for Patients with Locoregionally Advanced Head and Neck Cancer with a Contraindication to Cisplatin

**DOI:** 10.1007/s11864-023-01051-w

**Published:** 2023-01-25

**Authors:** Sangwoo S. Kim, Hannah C. Liu, Loren K. Mell

**Affiliations:** Department of Radiation Medicine and Applied Sciences, University of California San Diego, 3855 Health Sciences Drive, MC0843, La Jolla, CA 92093 USA

**Keywords:** Head and neck cancer, Radiation therapy, Chemotherapy, Cisplatin-ineligible

## Abstract

Significant advancements have been made in the treatment of locally advanced head and neck cancer, predominantly driven by the integration of concurrent chemotherapy with radiation therapy as a standard of care for many patients. The most heavily investigated chemotherapeutic is cisplatin, yet many patients are ineligible for cisplatin due to the presence of pre-existing medical comorbidities. Moreover, given the toxicity profile of cisplatin, identifying which patients stand to benefit from cisplatin is challenging, which is particularly evident in older patients. Efforts to better risk-stratify patients based on age, performance status, and the degree of pre-existing comorbidities are ongoing and have been increasingly utilized in national clinical trials. In parallel, exploration into alternative systemic agents, including novel targeted therapies and immunotherapies, in cisplatin-ineligible patients are rapidly expanding. Cumulatively, identifying appropriate treatment paradigms in patients who harbor contraindications to cisplatin can not only improve clinical outcomes but also critically mitigate detrimental adverse effects.

## Introduction

The treatment of head and neck cancer (HNC) has greatly advanced over the past two decades. Advancements in radiation therapy (RT) and surgical technique modalities have sought to decrease the significant treatment-associated morbidity [1, 2]. In parallel, multi-modality treatment integrating systemic agents with RT has undoubtedly improved patient outcomes. With the meta-analysis of chemotherapy in head and neck cancer (MACH-NC) demonstrating an overall survival (OS) benefit with concurrent RT with chemotherapy, particularly platinum-based agents such as cisplatin, combination treatment became the standard-of-care for many patients [3].

However, many patients may be ineligible for cisplatin due to age or medical comorbidities, or develop intolerance necessitating treatment breaks, or survive with chronic adverse effects [4]. In light of the increased rates of significant adverse effects of concurrent chemotherapy and radiation therapy (CRT), the MACH-NC study found a decreasing benefit of systemic therapy with increasing age. Even in highly selected patients who are deemed to have “low-risk” HNC, the incremental benefit of cisplatin over alternative systemic therapies and RT alone has been illustrated repeatedly [5–8]. One recent exception to this is in low-risk nasopharyngeal carcinoma, where treatment with intensity-modulated radiation therapy (IMRT) alone was not inferior to combined IMRT and cisplatin [9]. An in-depth review of treatment de-escalation studies is outside of the scope of this manuscript and has been reviewed elsewhere [10].

Whereas cisplatin is a clear standard-of-care treatment option for patients with locally advanced head and neck cancer (LA-HNC), a conundrum that oncologists face is in the management of patients who have contraindications to cisplatin. For an excellent clinically oriented discussion of this topic, we direct readers to Jhawar et al. [11•] Here, we present first a discussion on the consideration of age and medical comorbidities as they relate to cisplatin ineligibility, and conclude with a highlight of ongoing clinical trials investigating novel systemic agents to be used in conjunction with RT.

## Cisplatin ineligibility and toxicities

Patients who are deemed ineligible for cisplatin can be broadly divided into those who harbor an absolute contraindication to cisplatin and those whose age and medical comorbidities decrease the marginal benefit of concurrent cisplatin (Table 1). Given the abundance of clinical trials studying cisplatin-based CRT in HNC, the adverse effect profile and long-term effects of definitive therapy on a patient’s quality of life are well-documented [12, 13]. During treatment, cisplatin can cause nausea, vomiting, dysphagia, nephrotoxicity, ototoxicity, neuropathy, and hematologic toxicities. While nausea and vomiting are often self-limiting, reversible, and have readily available medications that can address these symptoms, the remainder of these adverse effects can be substantially more challenging to manage.

**Table 1:** Absolute and relative contraindications to cisplatin therapy. Select comorbidity indices used in recent trials are included

Absolute contraindications	Relative contraindications
Creatinine clearance < 50–60 mL/min	Advanced age (> 70)
Grade ≥ 2 tinnitus or pre-existing hearing loss	ECOG Performance Status score ≥ 2
Grade ≥ 2 neuropathy	Weight loss > 10% of baseline body weight in preceding 6 months
Hypersensitivity to platinum-based therapies	Low BMI < 16 kg/m^2^ or body weight (< 30 kg)
Pregnancy or lactation	Charlson Comorbidity Index ≥ 1*
HIV/AIDS with CD4 count < 200 μL	ACE-27 Index ≥ 1^†^
	HNCIG Omega Score < 0.80^‡^
	G-8 Score ≤ 14^‡^
	CARG Toxicity Score ≥ 30%^§^
	CIRS-G Score ≥ 4^#^

*BMI*, body mass index; *Modified Charlson Comorbidity Index: predicts 10-year survival using age (binned) and the presence/absence of cardiac, neurologic, pulmonary, gastrointestinal, endocrinologic, renal, and oncologic diagnoses. ^†^Adult Comorbidity Evaluation-27 (ACE-27): evaluates the presence and severity of cardiovascular, respiratory, gastrointestinal, endocrinologic, neurologic, psychiatric, rheumatologic, immunologic, and oncologic diagnoses in additional substance abuse and BMI. ^‡^Head and Neck Cancer Intergroup (HNCIG) omega score: predicts the overall event risk attributable to cancer events (as opposed to competing non-cancer mortality) based on age, performance status, sex, tumor site, T and N stage, p16 status, and smoking history. ^‡^Geriatric 8 (G-8): identifies older patients who could benefit from comprehensive geriatric assessment based on food intake, weight loss, mobility, neuropsychologic problems, BMI, number of prescription drugs, and self-perception of health. ^§^Cancer and Aging Research Group (CARG) Toxicity Score: estimates risk of severe chemotherapy-related side effects in older cancer patients based on age, cancer type, chemotherapy dose/number of agents, renal function, hearing, number of falls, and functional status. ^#^Cumulative Illness Rating Scale for Geriatrics (CIRS-G): quantifies burden of comorbid disease in older patients based on the presence and severity of cardiovascular, hematologic, respiratory, otolaryngologic, gastrointestinal, renal, genitourinary, rheumatologic, neurologic, endocrinologic, and psychiatric diagnoses. Ref: ***http://comogram.org***

A notable adverse effect of cisplatin is ototoxicity, with the two most common presentations being tinnitus and hearing loss. Few large studies have scrutinized patient-reported tinnitus. An institutional study of 22 patients utilizing a tinnitus handicap inventory suggested that the majority of patients who reported tinnitus had slight-to-mild symptoms [14]. Nonetheless, further data quantifying tinnitus is needed to identify mitigation strategies. Comparatively, there is more information regarding effects of CRT on hearing loss. Both RT and cisplatin can independently lead to hearing loss, which can be compounded by age-related hearing loss [15]. Unsurprisingly, hearing loss in older patients has been significantly associated with decreased quality of life [16]. The precise mechanism underpinning cisplatin-induced ototoxicity is unclear, but may be damage to hair cells in the cochlea and vascular epithelium of the cochlear walls [17]. Pharmacokinetic studies have found that cisplatin is retained in the cochlea, particularly in areas responsible for maintaining endolymph, for months to years following initial administration, which could successively lead to ongoing DNA damage and generation of reactive oxygen species [18, 19]. This suggests that interventions to increase clearance of cisplatin following treatment may lessen chronic ototoxicity. Given heterogeneity in routine evaluation of hearing prior to and following definitive CRT, the incidence and duration of hearing loss ranges widely, and also demonstrates variability across hearing thresholds, which have made precise quantification of the relative contributions of each therapy to hearing loss challenging [20]. Prior dosimetric analyses have suggested a dose-dependent relationship between radiation and degree of sensorineural hearing loss, but avoidance of the key structures responsible for hearing, in particular the cochlea, is frequently infeasible given the proximity between these structures and radiation targets [21].

Nephrotoxicity, while not as anatomically relevant in the treatment of HNC, is nonetheless critical to evaluate given the high prevalence of comorbidities, such as diabetes mellitus and hypertension, that increase rates of chronic kidney disease (CKD) in older patients with cancer [22]. In fact, rates of stage 3 or higher CKD in patients with solid tumors range between 12 and 25%, and these patients were shown to have an increased of death [22]. This may be due to limited access to nephrotoxic chemotherapeutics such as cisplatin, and this has been particularly salient in the treatment of muscle-invasive bladder cancer (MIBC) where baseline renal dysfunction is more common; for an excellent discussion of cisplatin eligibility in patients with MIBC, we refer to the review by Jiang et al. [23]

There are several medical scenarios in which cisplatin would not be routinely recommended, as summarized in Table 1. In many of these scenarios, patients would benefit from intensified therapy (over RT alone) with an alternative systemic agent, which is generally dependent on the treating physician, as there has not yet to be established a clearly superior alternative to cisplatin. In cases where the extent of pre-existing comorbidities is insufficient to withhold cisplatin completely, one option is to use weekly cisplatin at a lower dose. A retrospective analysis comparing 3-weekly (100 mg/m^2^) or weekly (40 mg/m^2^) dosing schedules suggested similar PFS between the schedules, but less nephrotoxicity with weekly cisplatin [24]. In the postoperative setting, JCOG 1008, a phase II/III trial comparing the aforementioned cisplatin schedules, demonstrated non-inferiority of weekly cisplatin along with decreased rates of grade 4 toxicities [25••]. The ongoing NRG-HN009 trial will inform the community on not only the relative effectiveness but also the adverse effect profiles of these two cisplatin dosing regimens in the definitive setting [26]. Cumulatively, data on the toxicity profiles of weekly cisplatin dosing may alter which patients are eligible for cisplatin.

In the following sections, we will discuss *relative*, rather than absolute, contraindications to cisplatin, with a specific focus on age and the contribution of competing risks, particularly how various prognostic factors affect patients’ risk for non-cancer mortality and propensity to tolerate intensive therapy. We will also review data on the use of concurrent RT with cetuximab and non-cisplatin-based radiosensitizing traditional chemotherapeutics. To conclude, we present an overview of ongoing trials seeking to integrate novel therapies and the rationale behind these approaches.

## Age as a relative contraindication

In the MACH-NC analysis, there was a clear differential benefit of chemotherapy as a function of age, with a less dramatic treatment effect in patients older than 70 [3]. Analysis of the Longitudinal Oncology Registry of Head and Neck (LORHAN) revealed that only 62% of patients received cisplatin either as monotherapy or in combination with other agents for systemic therapy, reflecting that a significant proportion of the population are deemed to be poor candidates for cisplatin, even if these therapies are medically indicated for them [27, 28]. Yet, age is unlikely to be useful as a sole predictor of treatment effectiveness, as age is highly correlated with the presence of medical comorbidities, which is independently related to clinical outcomes in patients with HNC [29]. This presents a complex question: At what point is cisplatin no longer indicated in an older patient population? Undoubtedly, there is no obvious threshold age at which cisplatin should be withheld, and so a more nuanced discussion on risk at the individual level is warranted.

Particularly salient for older patients is that they are frequently underrepresented in large clinical trials. Correspondingly, there is a paucity of data to guide firm treatment decisions. Despite the fact that individuals over the age of 70 constitute nearly 50% of the population of patients with cancer, an analysis by Hutchins et al. found that they represent just 13% of the population enrolled on large clinical trials [30, 31]. A study by Kish et al. found only 12% of locoregionally advanced HNC patients enrolled to NRG/RTOG trials were at least 70 years old; not surprisingly, these patients older than 70 had worse hematologic and renal toxicity, and OS, but similar locoregional control [31].

While radiation itself has many known adverse effects, in the era of increasing utilization of intensity-modulated RT with image guidance, older patients are able to complete courses of definitive RT. [32] Therefore, efforts have been focused on the use of systemic therapies. Prior analyses on patterns of failure illustrated that a significant proportion of deaths in clinical trials are attributable to comorbid illnesses, secondary malignancies, or treatment-related toxicities [3, 39]. A study by O’Neill et al. reported increased rates of hospitalization, ER visits, and feeding tube placement in older patients treated with CRT as compared to RT alone [33]. Given that delays in treatment are known to compromise oncologic outcomes, especially in HNC, pursuing intensive therapies only to encounter treatment-related toxicities may have diminishing benefits [34, 35]. Despite this, a patterns of care study utilizing the National Cancer Data Base (NCDB) demonstrated an increase in the use of concurrent systemic therapy from 64% in 2004 to 86% in 2012 [36].

Central to this is that older patients on average have more medical comorbidities, which places them at higher risk for death from non-cancer causes [29]. There are many validated tools used to quantify the degree of medical comorbidities or frailty, as described in Table 1. A study by Vitzthum et al. examined several indices in patients with head and neck cancer to develop a web-based application for risk-stratifying patients beyond traditional measures such as Eastern Cooperative Oncology Group (ECOG) performance status [37], on the premise that with increased competing risks, there is a smaller marginal benefit to intensive therapy. While smoking and alcohol consumption are known etiologies of HNC, these factors also increase frequencies of medical comorbidities that are often unable to be controlled for in statistical analyses [38]. Therefore, routinely used statistical analyses to investigate the effect of an intervention on composite endpoints, such as overall survival or recurrence-free survival, may lead to sub-optimal risk-stratification [39, 40]. Therefore, it is critical that the relative benefit of treatment intensification, in this case the addition of concurrent chemotherapy to RT, is not outweighed by concomitant increases in the incidence of competing events.

A population-based analysis of non-metastatic HNC from the SEER registry showed that patients could be effectively stratified according to relative risk of primary cancer recurrence vs. competing mortality using standard competing risk models [41]. More recent studies have used generalized competing event (GCE) regression techniques to estimate the effects of multiple risk factors on the relative hazards for cancer recurrence vs. competing mortality, or omega ratio [42]. In this framework, patients with a higher risk for cancer recurrence *relative* to competing mortality are theoretically more likely to benefit from treatment intensification. Thus, the omega ratio acts as both a prognostic *and* predictive modifier.

For example, this approach was used in an analysis of stage III-IVB HNC patients treated on three clinical trials (RTOG 9003, 0129, 0522) to develop a risk score (omega score) that effectively separated patients according to relative event hazards. Patients with an omega score ≥ 0.80 were found to benefit more from intensive treatment, as predicted by the model. This tool was further validated on the Meta-Analysis of Radiotherapy in Squamous Cell Carcinomas of Head and Neck (MARCH) and MACH-NC datasets, showing an interaction between the effect of intensive treatments (altered fractionation and concurrent chemotherapy with RT) based on the omega score, which depends on age, sex, performance status, tumor site, T and N category, p16 status, and smoking history [43]. Importantly, there was no clear benefit to treatment intensification for those with omega score < 0.80, in contrast to patients with omega scores ≥ 0.80.

Interestingly, in the meta-analysis utilizing the MARCH and MACH-NC data sets, patients who were predicted to have the highest PFS (best prognosis) appeared to derive the *most* benefit from intensive therapy. In other studies, omega scores have been particularly effective in stratifying patients over the age of 70, and this tool has since been incorporated into eligibility criteria for clinical trials in patients with a contraindication to cisplatin, including NRG-HN004 and NRG-HN008. This tool is also publicly available through the web-based application, http://www.comogram.org [42, 44]. In summary, while age is highly correlated with the presence of medical comorbidities that reduce the marginal benefit of treatment intensification, older patients with a low risk of non-cancer-related deaths can still derive a significant benefit to more aggressive therapies.

## Cetuximab as concurrent therapy

Cetuximab, a monoclonal antibody targeting the epidermal growth factor receptor (EGFR), was heavily investigated as an agent to be used concurrently with RT. The rationale for its application was based on studies that showed abnormal expression of EGFR in many epithelial malignancies, including HNC [45]. Further, increased expression of EGFR has been correlated with resistance to ionizing radiation, and EGFR inhibition with cetuximab was thought to sensitize cells to RT. [46]

A landmark study published by Bonner et al. combining cetuximab and RT found improved outcomes with the addition of cetuximab to RT, with increased progression-free survival (PFS) and OS (55% with cetuximab + RT versus 45% with RT alone, *p* = 0.05) [47]. However, only approximately 26% of the patients were ≥ 65 years old, with over 85% having Karnofsky performance score ≥ 80, reflecting a relatively young patient population with favorable performance status. In a post hoc subgroup analysis of this study, there was no clear benefit to cetuximab in patients older than 65 or with poorer performance status [48]. Nevertheless, cetuximab has been increasingly used in older patients, driven in part by its known tolerability [49]. In a study on patterns of care in older patients, Baxi et al. revealed marked increases in the use of concomitant systemic therapies with RT, with cetuximab representing the most common choice [50].

However, it is important to note that the overall toxicity of concurrent RT with cetuximab is not necessarily less as compared to treatment with cisplatin, as shown in the RTOG 1016 [5], De-ESCALaTE HPV [6], TROG 12.01 [8], and ARTSCAN III [51] trials. Each of these trials had slight differences in RT administration (total dose and fractionation patterns) and cisplatin administration (weekly low-dose cisplatin versus high-dose cisplatin every 3 weeks for 2–3 cycles), but cumulatively demonstrated no significant difference in the incidence of severe toxicities. These trials revealed how the two regimens have different adverse effect profiles, with greater hematologic and renal toxicities with cisplatin, but more frequent dermatologic toxicities with cetuximab. Critically, each of these trials demonstrated inferior outcomes with cetuximab compared to cisplatin. Therefore, while cetuximab would not be appropriate in a cisplatin-eligible patient, depending on a patient’s specific comorbidities, it remains a reasonable alternative in patients with contraindications to cisplatin.

Currently, there is a scarcity of randomized published data on comparative effectiveness of systemic agents in patients who are ineligible or are poor candidates for cisplatin. In the following sections, we will highlight three categories of agents: (1) cytotoxic radiosensitizers, (2) immune checkpoint inhibitors (ICIs), and (3) novel targeted therapies.

### Cytotoxic radiosensitizers

Three commonly utilized chemotherapeutic alternatives to cisplatin are (1) carboplatin and 5-fluorouracil (5-FU), (2) carboplatin and paclitaxel, and (3) docetaxel. One of the initial CRT studies, GORTEC 94-01, explored combined carboplatin and 5-FU and found improved OS, albeit with higher, not statistically significant, occurrence of grade 3+ adverse effects (56% with CRT vs. 30% in RT alone) [52]. GORTEC 99-02, which studied both the addition of chemotherapy (carboplatin and 5-FU) and accelerated fractionation (6 fractions weekly and twice daily treatment), found that the most favorable outcomes were with the addition of chemotherapy [53]. A large phase III trial from Tata Memorial Hospital (DHANUSH) recently investigated weekly concurrent docetaxel with RT versus RT alone in 356 patients [54•]. This study reported a significant improvement in 2-year DFS (30.3% in RT alone versus 42% with RT plus concurrent docetaxel) and 2-year OS (41.7% in RT alone versus 50.8% with RT plus concurrent docetaxel). The authors noted higher incidences of grade ≥ 3 adverse effects with concurrent docetaxel as compared to RT alone, highlighting the need for proper patient selection.

It is unclear which alternative regimen, if any, is superior, since there are no randomized trials yet comparing these regimens to each other in the cisplatin-ineligible population. A comparative effectiveness analysis in US veterans with head and neck cancer found that carboplatin-based regimens were associated with superior outcomes compared to cetuximab, after controlling for confounding using propensity score modeling; this effect was mostly driven by patients with oropharyngeal cancer [55]. This study was limited by missing data on HPV status and additional key clinical characteristics, such as smoking and performance status, however. A separate analysis of the SEER-Medicare database similarly suggested that carboplatin-based regimens were not only superior to cetuximab, but were also similar in efficacy to cisplatin [56]. Unfortunately, selection bias is a common problem with retrospective studies in competing risks populations, even after controlling for confounding variables with propensity score models [57]. Collectively, these studies highlight the need to compare alternative treatment regimens in randomized trials.

### Immune checkpoint inhibitors

Perhaps the most prominent class of therapies in oncology over the past decade is immune checkpoint inhibitors (ICIs), the most prevalent being monoclonal antibodies targeting the PD-1:PD-L1 axis. Data from patients with recurrent or metastatic HNSCC after platinum chemotherapy revealed marked improvements in overall survival with nivolumab and pembrolizumab, both of which target PD-1 [58, 59]. Therefore, there has been great enthusiasm in integrating ICIs in the definitive setting. One of the earliest reported large trials was JAVELIN Head and Neck 100, a phase 3 trial evaluating CRT with bolus cisplatin and concurrent avelumab (an anti-PD-L1 monoclonal antibody) with maintenance avelumab for up to 12 months, for patients with previously untreated LA-HNC [60]. JAVELIN ultimately did not demonstrate improved PFS in the experimental arm [60]. The KEYNOTE-412 trial adopted a similar schedule, but using pembrolizumab (anti-PD1) instead of avelumab (anti-PDL1) [61]. Preliminary data presented in abstract form indicated a trend towards improved event-free survival with pembrolizumab, particularly among patients with combined positive score (CPS) ≥ 1, but the improvements were not statistically significant [61].

An active question is whether ICIs could be used *instead* of cisplatin. We highlight two angles in which the use of ICIs in the definitive setting is ongoing. The first approach takes advantage of inherent differences in the underlying biology of HPV-associated HNC, which is known to have a distinct tumor microenvironment with increased infiltration of immune cells [62]. In particular, two ongoing clinical trials are comparing cisplatin and RT vs. RT with ICIs for selected patients with p16-positive HNC: the phase II/III NRG-HN005 trial [63] and the phase II randomized KEYCHAIN trial [64], which target favorable-risk and unfavorable-risk p16-positive HNC, respectively. NRG-HN005 is a three-arm trial comparing standard dose RT (70 Gy) plus bolus cisplatin vs. reduced dose RT (60 Gy) plus bolus cisplatin, to reduced dose RT plus concurrent and adjuvant nivolumab (6 cycles total every 2 weeks up to 6 weeks post-RT) [63]. KEYCHAIN is a two-arm trial comparing standard dose RT (70 Gy) plus bolus cisplatin vs. standard dose RT plus concurrent and adjuvant pembrolizumab (20 cycles total every 3 weeks up to 1 year post-RT) [64]. Data from these trials may help determine whether ICIs are a reasonable alternative to cisplatin in selected populations.

Another application of ICIs has been for patients unable to receive cisplatin due to medical contraindications. Three trials (PembroRad [65•], GORTEC-REACH [66], and NRG-HN004 [67]) have studied ICIs in patients unfit for cisplatin. Notably, RT with cetuximab was the control arm in each of these trials. The PemboRad trial used pembrolizumab concurrently with RT (but no maintenance therapy) in the experimental arm, while GORTEC-REACH used avelumab plus cetuximab with RT, and NRG-HN004 used durvalumab (a PD-L1 inhibitor) with RT. These trials have provided critical data on the comparative effectiveness of ICIs to cetuximab (Table 2) [65•, 66, 67]. While the PembroRad trial did not find significant differences between the two arms, acute toxicity was lower with pembrolizumab (overall grade ≥ 3 toxicity of 74% in pembrolizumab arm versus 92% in cetuximab arm) [65•]. In the GORTEC-REACH trial (cisplatin-unfit cohort), although PFS was nominally improved with avelumab, the difference was not statistically significant [66]. In NRG-HN004, patients receiving durvalumab had statistically significantly worse locoregional control, with a non-significant trend towards worse PFS, albeit with numerically lower rates of grade ≥ 3 toxicities [67]. These studies and downstream translational research initiatives have the potential to determine which patients may benefit from ICIs, and in turn, redefine the management of cisplatin-ineligible patients with HNC.

**Table 2:** Summary of recently reported trials studying immune checkpoint inhibitors in patients with locally advanced head and neck cancer who have a contraindication to cisplatin

	Treatment arms	Clinical outcomes (Arm 1 vs. Arm 2)	Toxicity
PembroRad [65•]	**Arm 1**: Concurrent cetuximab + RT **Arm 2**: Concurrent pembrolizumab + RT	**15-month LRC**: 59% vs. 60% (*p* = 0.91) **2-year PFS**: 39.9% vs. 42.4% (*p* = 0.47) **2-year OS**: 55.3% vs. 61.7% (*p* = 0.49)	Grade ≥3 AE: 92% vs. 74% (*p* = 0.006)
GORTEC-REACH (cisplatin-ineligible cohort) [66]	**Arm 1**: Concurrent cetuximab + RT **Arm 2**: Concurrent avelumab + RT followed by adjuvant avelumab	**2-year PFS**: 31% vs. 44% (*p* = 0.15) **2-year OS**: 54% vs. 58% (*p* = 0.69) **2-year LRF**: 44% vs. 34% (*p* = 0.34)	Grade ≥ 3 AE: 80% vs. 80% (*p* = 0.91)
NRG-HN004 [67]	**Arm 1**: Concurrent cetuximab + RT **Arm 2**: Concurrent durvalumab + RT followed by adjuvant durvalumab	**2-year PFS**: 66% vs. 51% (*p* = 0.92) **2-year OS**: 78% vs. 70% (*p* = 0.72) **2-year LRF**: 15% vs. 32% (*p* = 0.04)	Grade ≥ 3 AE: 79% vs. 69% (*p* = NS)

*RT*, radiation therapy; *LRC*, locoregional control; *LRF*, locoregional failure; *PFS*, progression-free survival; *OS*, overall survival; *AE*, adverse events

### Novel targeted therapies

**Fig. 1:**
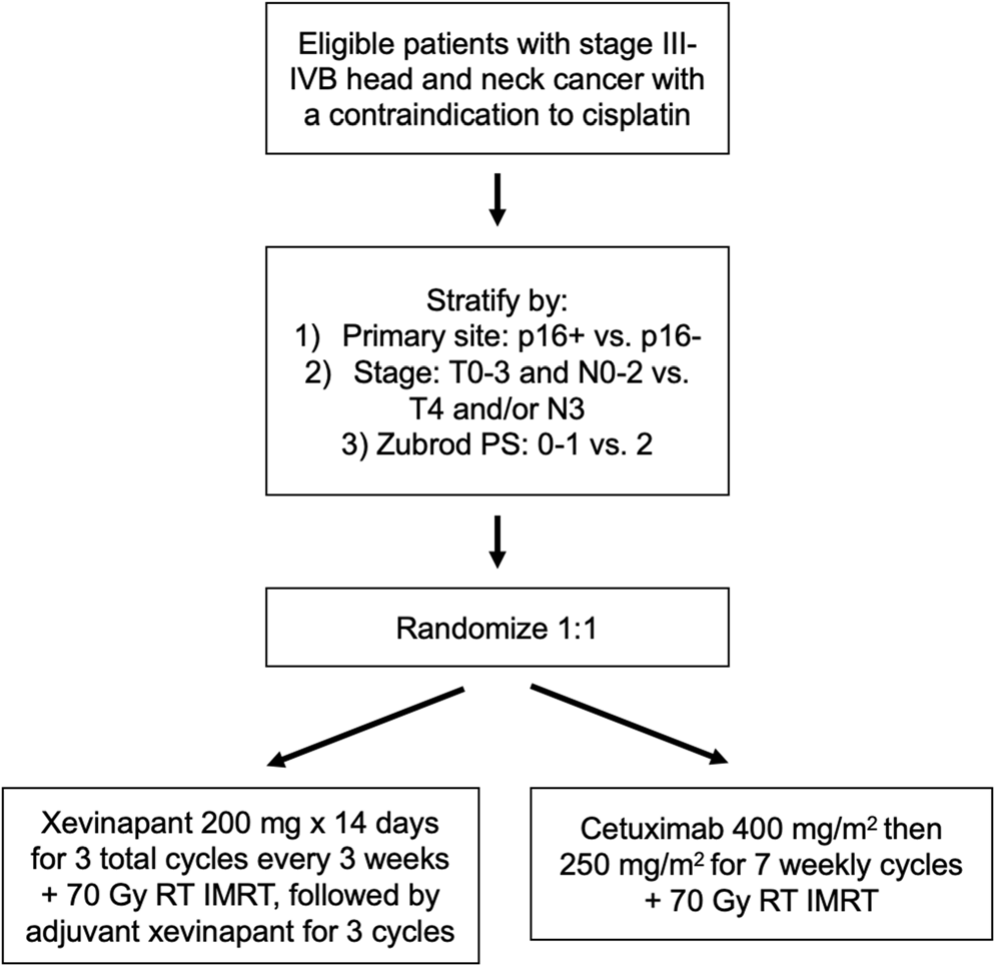
Schema of upcoming NRG-HN012 trial to compare RT with concurrent and adjuvant xevinapant, a second mitochondria-derived activator of caspases (SMAC) mimetic, vs. RT with concurrent cetuximab in patients with locally advanced head and neck cancer who have a contraindication to cisplatin.

Increasingly, targeted therapies exploiting novel pathways are being studied as substitutes for cisplatin in combination with RT. An ongoing phase I trial (NRG-HN008), is evaluating the use of peposertib, a DNA-dependent protein kinase (DNA-PK) inhibitor, with RT in LA-HNC patients with a contraindication to cisplatin [68]. Preclinical data have demonstrated that this agent suppresses repair of radiation-induced DNA double-strand breaks, increases PD-L1 expression on cancer cells, and enhances inflammatory signaling [69]. Another class of agents includes second mitochondria-derived activator of caspases (SMAC) mimetics, which antagonize apoptosis inhibitor proteins (IAPs) and exhibit radiosensitizing effects via caspase-dependent and CD8 T cell–dependent pathways [70••]. A phase 2 study by the GORTEC investigated one such agent, xevinapant, in combination with standard, high-dose cisplatin for the treatment of locally advanced HNC and demonstrated significantly improved 5-year OS (53% with CRT and xevinapant vs. 28% with CRT alone) [71]. Ongoing and recently initiated randomized trials, including the upcoming NRG-HN012 trial specifically in cisplatin-ineligible patients (Fig. 1), will further assess the efficacy of this novel therapeutic class [72]. Studies such as these expand the set of anti-cancer therapeutics and provide opportunities to examine the effects of novel agents in combination with RT.

## Conclusion

Cisplatin remains the radiosensitizer of choice for the majority of patients with locoregionally advanced head and neck cancer. However, due to absolute or relative contraindications, many patients must receive alternatives to cisplatin, which continue to result in high rates of treatment failure. Further research to study novel strategies that may improve outcomes for these patients is thus needed. As results of studies with novel agents come available, treatment options for patients with a contraindication to cisplatin will ideally expand, along with improved outcomes, lower toxicity, and enhanced quality of life.
